# Molecular recognition using tetralactam macrocycles with parallel aromatic sidewalls

**DOI:** 10.3762/bjoc.15.105

**Published:** 2019-05-09

**Authors:** Dong-Hao Li, Bradley D Smith

**Affiliations:** 1 Department of Chemistry and Biochemistry, University of Notre Dame, 236 Nieuwland Science Hall, Notre Dame, Indiana 46556, United States

**Keywords:** fluorescent dye, host–guest chemistry, hydrogen bonding, hydrophobic effect, macrocycles, rotaxane, supramolecular

## Abstract

This review summarizes the supramolecular properties of tetralactam macrocycles that have parallel aromatic sidewalls and four NH residues directed into the macrocyclic cavity. These macrocycles are versatile hosts for a large number of different guest structures in water and organic solvents, and they are well-suited for a range of supramolecular applications. The macrocyclic cavity contains a mixture of polar functional groups and non-polar surfaces which is reminiscent of the amphiphilic binding pockets within many proteins. In water, the aromatic surfaces in the tetralactam cavity drive high affinity due the hydrophobic effect and the NH groups provide secondary interactions that induce binding selectivity. In organic solvents, the supramolecular factors are reversed; the polar NH groups drive high affinity and the aromatic surfaces provide the secondary interactions. In addition to an amphiphilic cavity, macrocyclic tetralactams exhibit conformational flexibility, and the combination of properties enables them to be effective hosts for a wide range of guest molecules including organic biscarbonyl derivatives, near-infrared dyes, acenes, precious metal halide complexes, trimethylammonium ion-pairs, and saccharides.

## Review

### 1. Introduction

A large fraction of host–guest chemistry research uses macrocyclic compounds as the host molecules [1–2]. There are several reasons for this circumstance. Macrocycles are often relatively easy to synthesize and they have inherently preorganized structures that favor high affinity and shape-selective association of guest molecules or ions. Usually, it is the solvent that determines which non-covalent interactions are the most important for strong association. Polar interactions are often dominant in weakly polar organic solvents, and thus the structure of an effective macrocyclic host must include polar functional groups. In water, association is often driven by the hydrophobic effect which means the interior cavity of an effective macrocyclic host should have sections with non-polar surfaces [3]. A macrocyclic host molecule with an amphiphilic cavity – that is, a cavity containing a mixture of polar functional groups and non-polar surfaces in close proximity – is likely to be a versatile receptor with the potential to bind guests in both non-polar organic solvents and water [4–13].

**Scheme 1:**
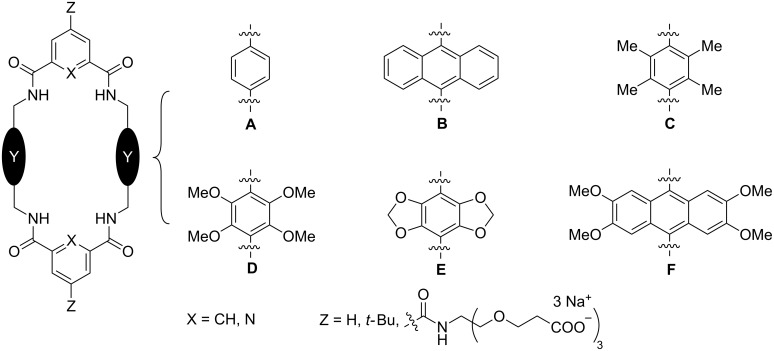
Chemical structures of the tetralactam host macrocycles that are covered by this review.

Macrocyclic tetralactams have been studied for several decades as hosts for various charged and neutral guest molecules [14–15], and in this review article, we focus on the specific group of tetralactam macrocycles shown in Scheme 1. These structures are all [2 + 2] macrocycles comprised of two 1,3-aryl dicarboxamide bridging units connected by two parallel aromatic sidewalls and we have chosen to highlight them as one of the few classes of macrocyclic host molecules that have an amphiphilic cavity. As described below, the range of guest structures that bind within the cavity of this host family is quite broad. In the following sections, we divide the guest structures into two large categories; those that associate with a tetralactam host in a reversible solution-state equilibrium and those that are permanently trapped inside a tetralactam macrocycle as an interlocked rotaxane or catenane. It is important to emphasize that the scope of the host structures in this review is intentionally quite focused and does not include tetralactams with angular aromatic sidewalls (studied by research groups led by Hunter [16], Vögtle [17], Schalley [18], and Chen [19]) or tetralactams with aliphatic sidewalls (studied by the groups led by Jurczak [15], Bowman-James [20], Lüning [21], and Thordarson [22]).

### 2. Macrocyclic structure and amphiphilic cavity

The peripherally appended Z groups on the bridging units in each macrocycle determine the solubility in different media without directly impacting the guest recognition within the central cavity. In organic solvents, hydrogen bonding with the four tetralactam NH residues is the dominant interaction that drives encapsulation of complementary guests, with aromatic stacking as a secondary contributor [23–25]. In water, the thermodynamic importance of these non-covalent factors is reversed; hydrogen bonding is relatively weak and strong guest association occurs when hydrophobic sections of a complementary guest are able to contact the hydrophobic interior surfaces of the two aromatic sidewalls [26].

**Figure 1:**
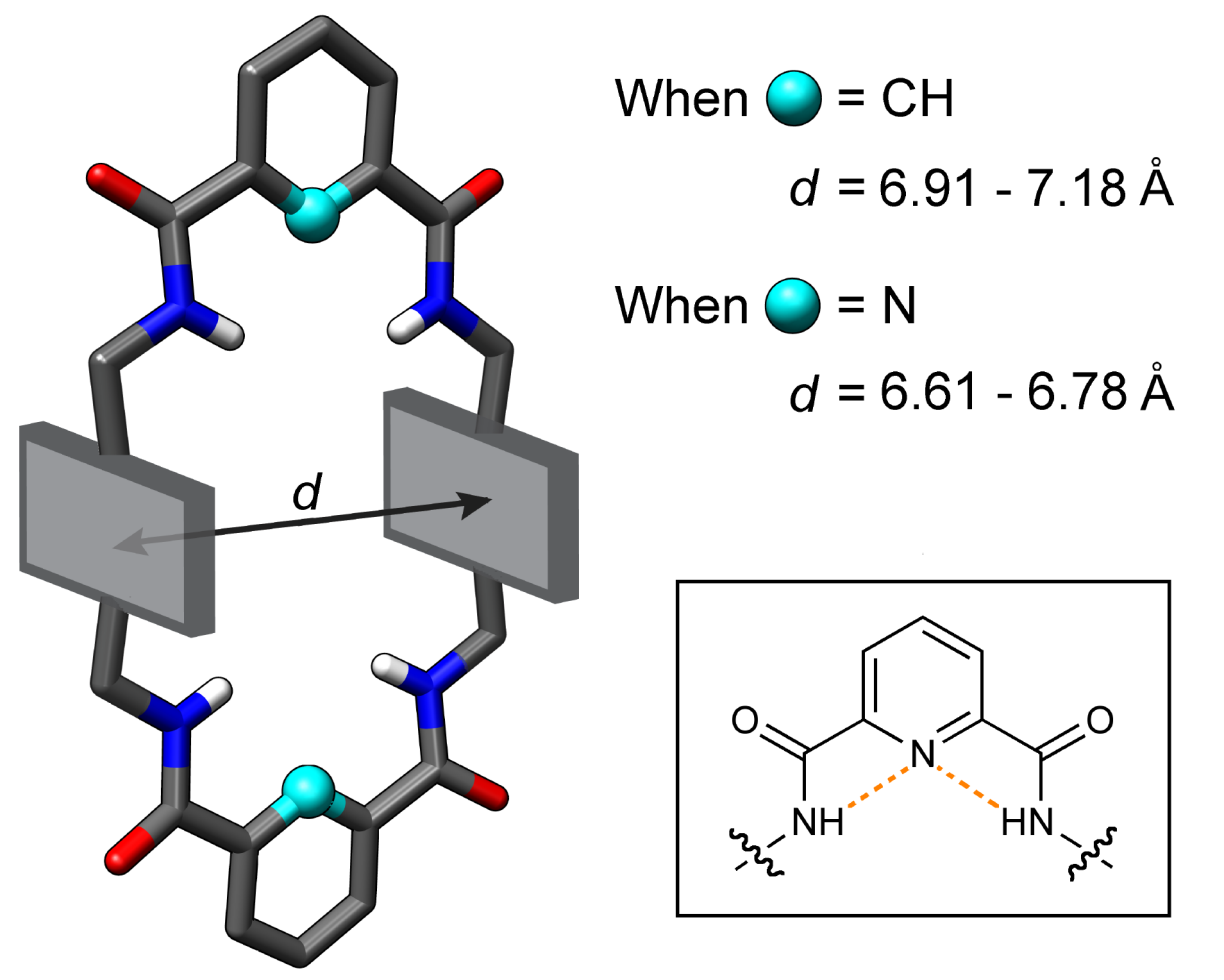
X-ray crystal structures of various squaraine rotaxanes show that the macrocycle bridging units control the distance *d* between the two cofacial aryl walls in the surrounding macrocycle. When the two bridging units are 2,6-pyridine dicarboxamides there is internal hydrogen bonding (see insert), which contracts the macrocycle compared to analogs with 1,3-benzene dicarboxamides as the two bridging units. Reprinted with permission from [31], copyright 2010, American Chemical Society.

The first macrocycle system in Scheme 1 to be studied in detail was tetralactam **A** reported by the Leigh group in the mid 1990s [27–28]. The macrocycle has two parallel 1,4-phenylene sidewalls and when the two bridging units are 1,3-benzene dicarboxamides (X = CH) there is considerable conformational flexibility. For example, the empty macrocycle can easily adopt a conformation with one or more of its NH residues directed out of the macrocycle cavity which enables intermolecular hydrogen bonding [29]. This promotes macrocycle self-aggregation and insolubility in nonpolar solvents (<1 mg L^−1^ in chloroform). There are two ways to reduce the flexibility and preorganize the macrocyclic structure in a stable conformation with all NH residues directed into the cavity. One way is to use 2,6-pyridine dicarboxamides as the bridging units which permits intramolecular hydrogen bonding within the cavity (Figure 1) [30]. This internal hydrogen bonding stabilizes conformations that have internally directed NH residues and it also contracts the macrocycle cavity. A measure of the cavity size is the centroid-to-centroid distance *d* between the two parallel aromatic sidewalls which can be obtained from X-ray crystal structures. As shown in Figure 1, *d* values for macrocycles with 2,6-pyridine dicarboxamide bridges are in the range of 6.61–6.78 Å, which is shorter than the range of 6.91–7.18 Å for analogous macrocycles with 1,3-benzene dicarboxamide bridges [31]. In some cases, it has been shown that the narrower cavity leads to slower rates of guest encapsulation but no significant changes in association constants [32].

**Figure 2:**
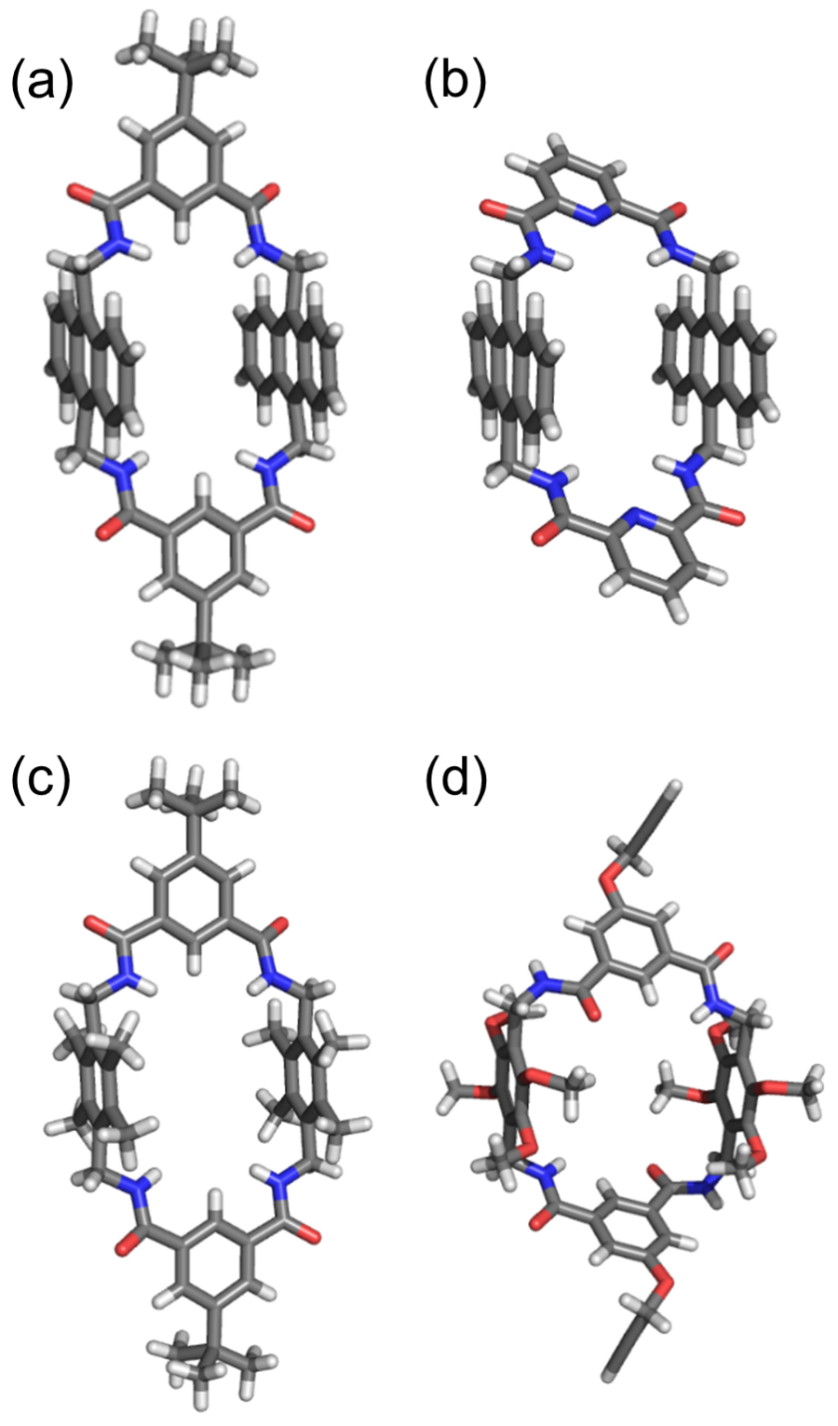
X-ray crystal structures of macrocycles that are representative of the tetralactam systems in Scheme 1. In (a) and (b) are examples of tetralactam **B** (9,10-anthrylene sidewalls) and in (c) is an example of **C** (2,3,5,6-tetramethyl-1,4*-*phenylene sidewalls) [23–25]. In each case, all NH residues are directed into the macrocycle cavity. In (d) is an example of tetralactam **D** (2,3,5,6-tetramethoxy-1,4*-*phenylene sidewalls), with two NH groups pointing out of the cavity [34]. Solvent molecules in these structures are omitted for clarity.

A second way to preorganize the macrocycle structure in a conformation with all NH residues directed into the macrocycle cavity was developed by the Smith group [33]. They substituted the 1,4-phenylene sidewalls with sterically encumbered aromatic units that reduced conformational flexibility. Two successful examples are macrocycles **B** and **C** with 9,10-anthrylene and 2,3,5,6-tetramethyl-1,4*-*phenylene sidewalls, respectively (Figure 2). X-ray crystal structures of these systems show all four NH residues directed into the cavity, even when the bridging unit is 1,3-benzene dicarboxamide (X = CH) and the cavity does not contain a high-affinity guest [23–25]. An exception with this sterically constrained preorganization strategy is macrocycle **D,** with 2,3,5,6-tetramethoxy-1,4*-*phenylene sidewalls, which adopts a *C*2-symmetric conformation with two NH groups pointing out of the cavity (Figure 2d) [34].

### 3. Guests of tetralactam macrocycles

#### 3.1. Biscarbonyls and close analogues

**Scheme 2:**
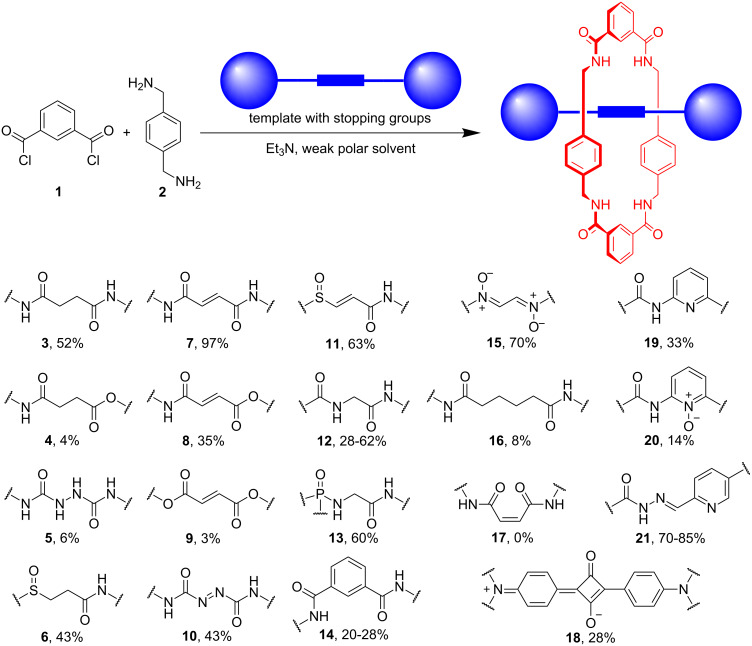
Synthetic yields of [2]rotaxanes with different dumbbell-shaped templates and tetralactam **A** as the surrounding macrocycle.

**Scheme 3:**
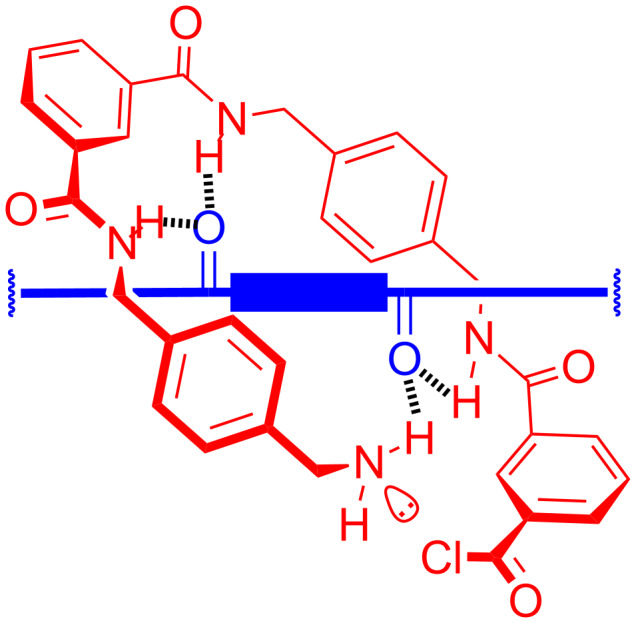
Supramolecular picture of the amide-bond-formation step that clips tetralactam **A** around a biscarbonyl template to form a [2]rotaxane.

The first attempt to prepare tetralactam **A** employed a chemical reaction that mixed isophthaloyl dichloride (**1**) and 1,4-xylylenediamine (**2**) [27]. This procedure only produced a small amount of the [2 + 2] macrocycle and a larger amount of the corresponding [2]catenane. Subsequent work found that conducting the reaction in the presence of a dumbbell-shaped template was a general way to make a wide range of [2]rotaxanes [28]. Listed in Scheme 2 are the yields of [2]rotaxane produced using various biscarbonyl-based templates [35–43]. These reactions are remarkable examples of templated syntheses since they form four covalent bonds and bring together five molecules in a single reaction. The key step is the final amide-bond formation that clips the tetralactam around the template (Scheme 3) [35]. The template molecule favors this step over the alternative intermolecular reaction that leads to larger oligomers. The poor solubility of **A** prevents the traditional host–guest affinity measurements; however, the [2]rotaxane yield is a useful indicator of template affinity for the internal cavity of the tetralactam precursor that is undergoing cyclization. Inspection of the yields in Scheme 2 shows that higher rotaxane yields are obtained when the template has: (a) preorganized shape (compare rigid fumaryl templates **7**, **8**, **9**, **10** and **11** with corresponding flexible succinyl analogues **3**, **4**, and **6**), (b) increased hydrogen-bonding-acceptor basicity which is obtained by replacing ester carbonyls with amide carbonyls (compare guest templates **7**, **8** and **9**), and (c) complementary distance between the two carbonyls (compare template **3** with **16**).

#### 3.2. Squaraine, thiosquaraine, croconaine, and acene guests

Rotaxane template **18** in Scheme 2 is a squaraine dye whose central core has two oxygen atoms that can form hydrogen bonds with the tetralactam NH residues. Squaraine rotaxanes were first prepared by the Smith group in 2005 using the Leigh-type clipping method [44]. As a general trend the photophysical property of a squaraine dye is improved when it is encapsulated by tetralactam **A**, **B**, or **C**, but squaraine fluorescence is quenched when the dye is encapsulated by tetralactam **E** whose structure has extremely electron-rich sidewalls [34].

**Figure 3:**
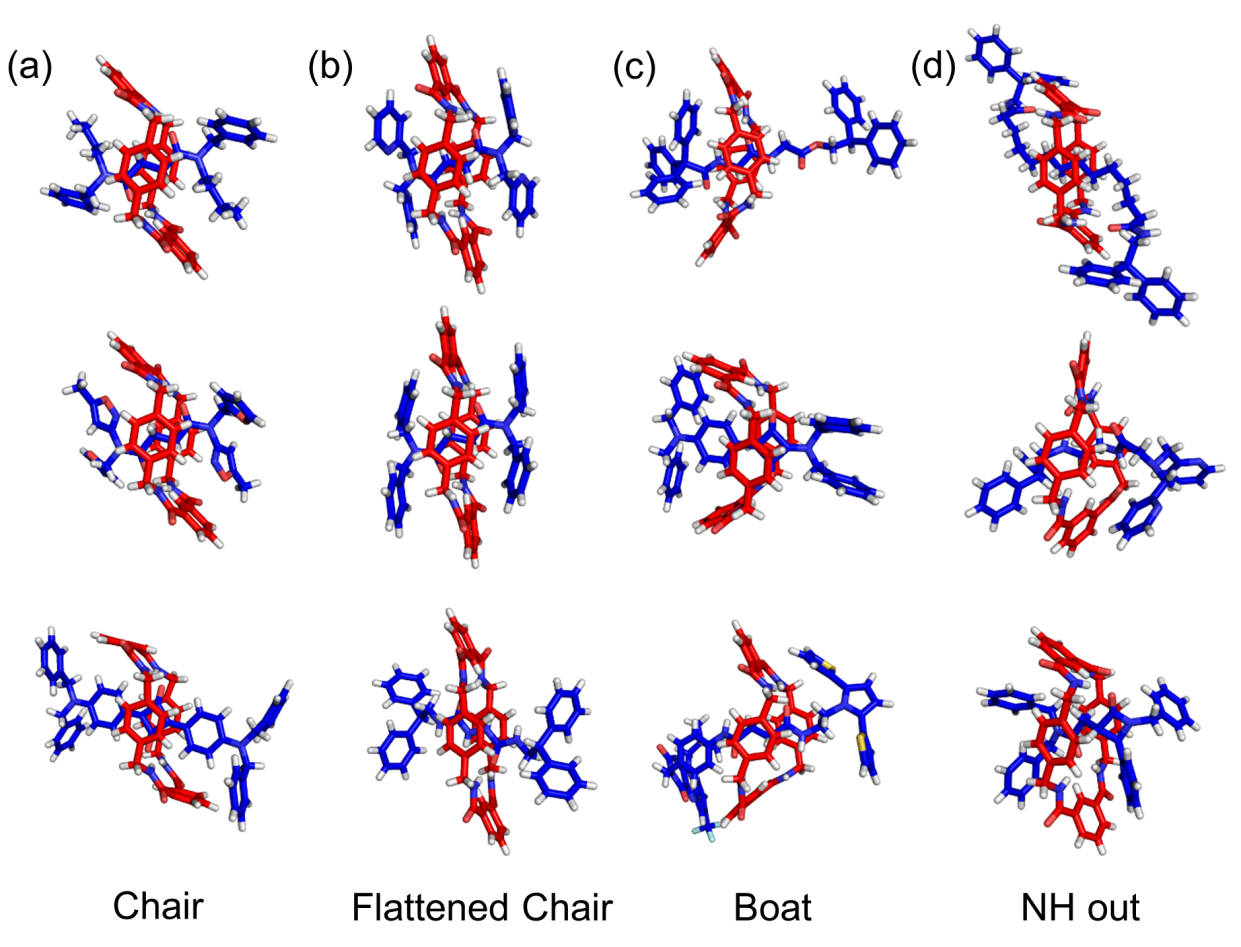
Selected X-ray structures of [2]rotaxanes with tetralactam **A** as the surrounding macrocycle reported by groups led by Leigh, Smith, Cooke, and Berná [37,39,50,52–56].

The moderate flexibility of tetralactam **A** is one of the reasons why it is able to accommodate quite a disparity of encapsulated guests [45–49]. In Figure 3 is a collection of different [2]rotaxane X-ray structures with tetralactam **A** as the surrounding macrocycle. The collection has been divided into four groups according to the macrocyclic conformation. The first three groups have all macrocycle NH residues directed inwards (which is favored when the macrocycle-bridging units are 2,6-pyridine dicarboxamides) with the macrocycle adopting a chair, flattened chair, or a boat conformation. The common theme of the fourth group of macrocyclic conformations is an outward directed NH residue. Solution-state NMR data suggests that the surrounding tetralactam in these [2]rotaxanes undergoes rapid exchange between these different conformations, a dynamic process that has been called macrcocyle breathing, and that there is also simultaneous co-conformational motion such as macrocycle pirouetting around the encapsulated guest [50–51].

**Figure 4:**
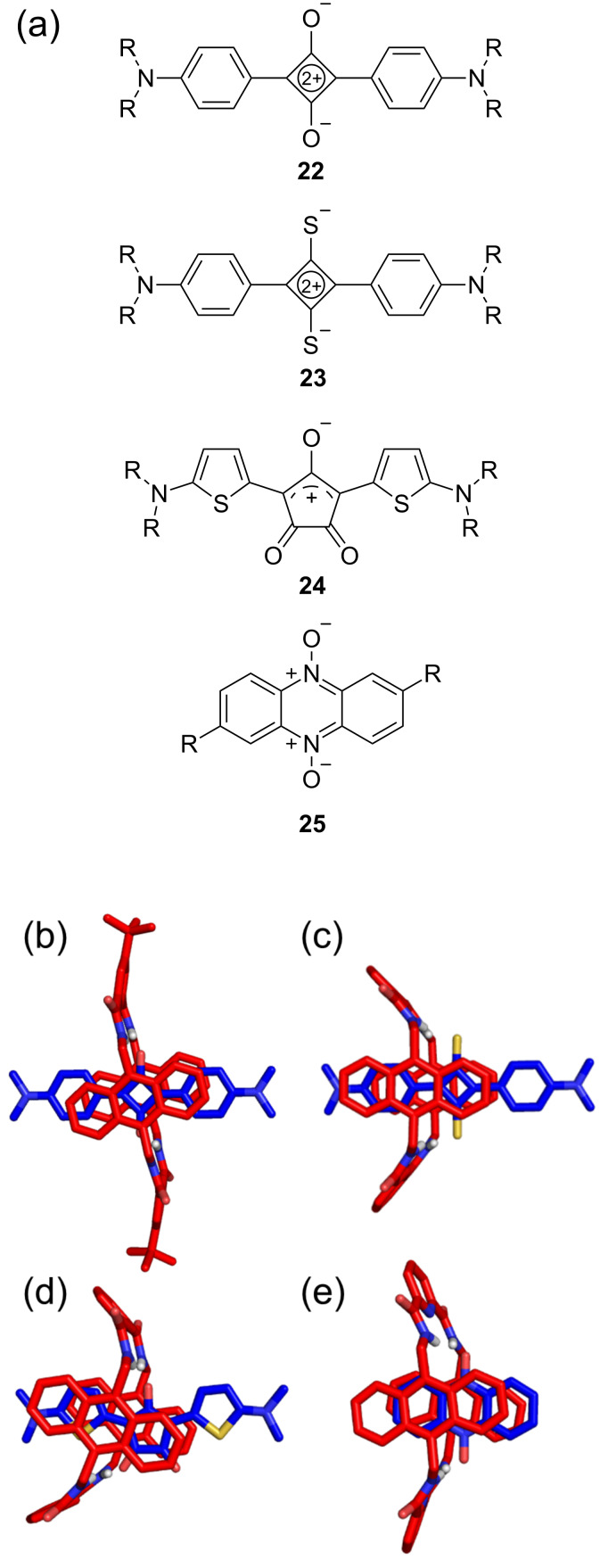
(a) Chemical structures of squaraine, thiosquaraine, croconaine, and acene guests that can bind inside tetralactam **B**. In (b) is an X-ray crystal structure of a complex comprised of a squaraine guest encapsulated by tetralactam **B** (X = CH, Z = *t*-Bu) in a flattened chair conformation [33]. In (c) and (d) are calculated structures (semiempirical, PM7) of complexes comprised of a thiosquaraine or croconaine, respectively, encapsulated by tetralactam **B** (X = CH, Z = *t*-Bu) in a boat conformation [57–59]. In (e) is an X-ray crystal structure of an azaacene guest encapsulated by tetralactam **B** (X = N, Z = H) in a boat conformation [25].

The Smith group has found that an organic soluble version of anthracene-containing tetralactam **B** is able to encapsulate squaraine, thiosquaraine and croconaine dyes [33,57–59]. Solid-state structures of various squaraine complexes show the surrounding macrocycle in a flattened chair conformation. In contrast, the surrounding macrocycle adopts a boat conformation in complexes that encapsulate larger thiosquaraine and croconaine dyes (Figure 4). A recent work by Mateo-Alonso and co-workers has reported several crystal structures of acene and azaacene guests inside tetralactam **B** with the surrounding macrocycle in chair or boat conformations [25].

**Scheme 4:**
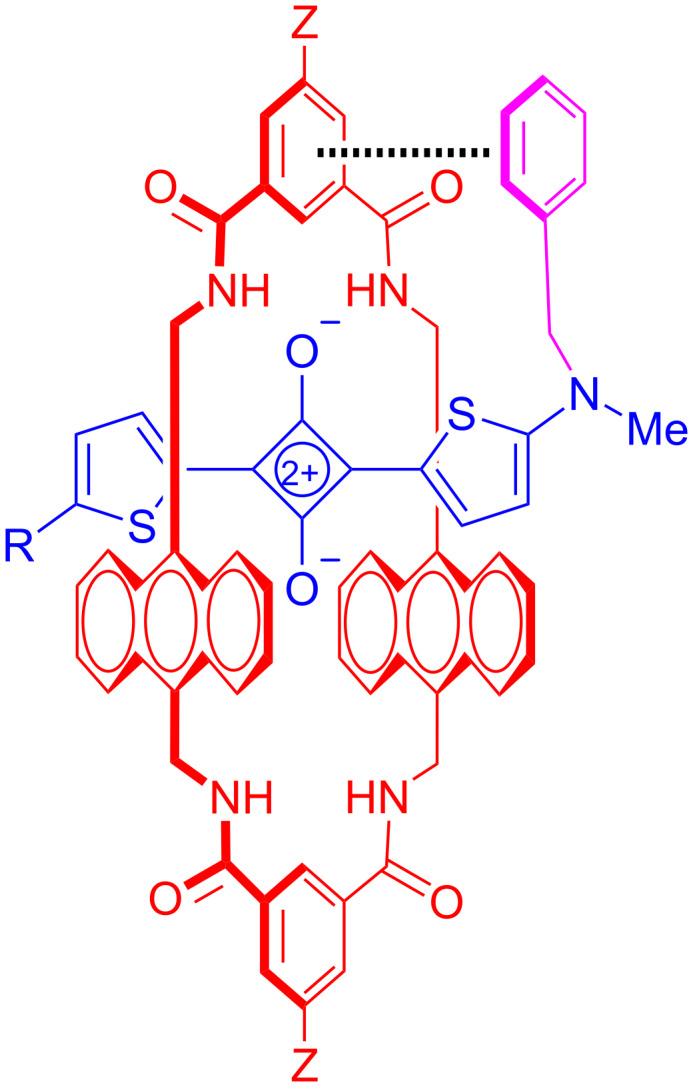
Complex stabilization due to guest back folding and aromatic stacking with the surrounding tetralactam macrocycle.

Recent work has shown that water-soluble versions of anthracene tetralactam **B** can be threaded by water soluble squaraine dyes with very high affinities (*K*_a_ ≈ 10^9^ M^−1^). The fact that tetralactams can strongly bind squaraine dyes both in organic solvent and in water reflects an advantage of the amphiphilic cavity. There is hydrogen-bonding capacity for squaraine binding in organic solvents and internal hydrophobic surfaces to drive binding in water. Solvent studies have found that squaraine affinities for tetralactam **B** are in the relative order of methanol < chloroform < water, reflecting the relatively large thermodynamic importance of the hydrophobic effect [60]. Interestingly, isothermal titration calorimetry studies revealed that the high squaraine binding in water is due primarily to a highly favorable enthalpic change [61]. Furthermore, tetralactam threading studies using water-soluble squaraine guests with flanking benzylic groups have produced *K*_a_ values that were close to 10^11^ M^−1^ [62]. The affinity gain is due to a guest back-folding effect where the affinity of the threaded squaraine is enhanced by additional contacts made by the dye’s flanking benzylic groups with the external surface of the surrounding macrocycle (Scheme 4). This type of noncovalent interaction between an encapsulated guest and the external surface of the surrounding tetralactam has been noted before as a factor that effects [2]rotaxane dynamics [56,63].

#### 3.3. Precious metals complexes

**Figure 5:**
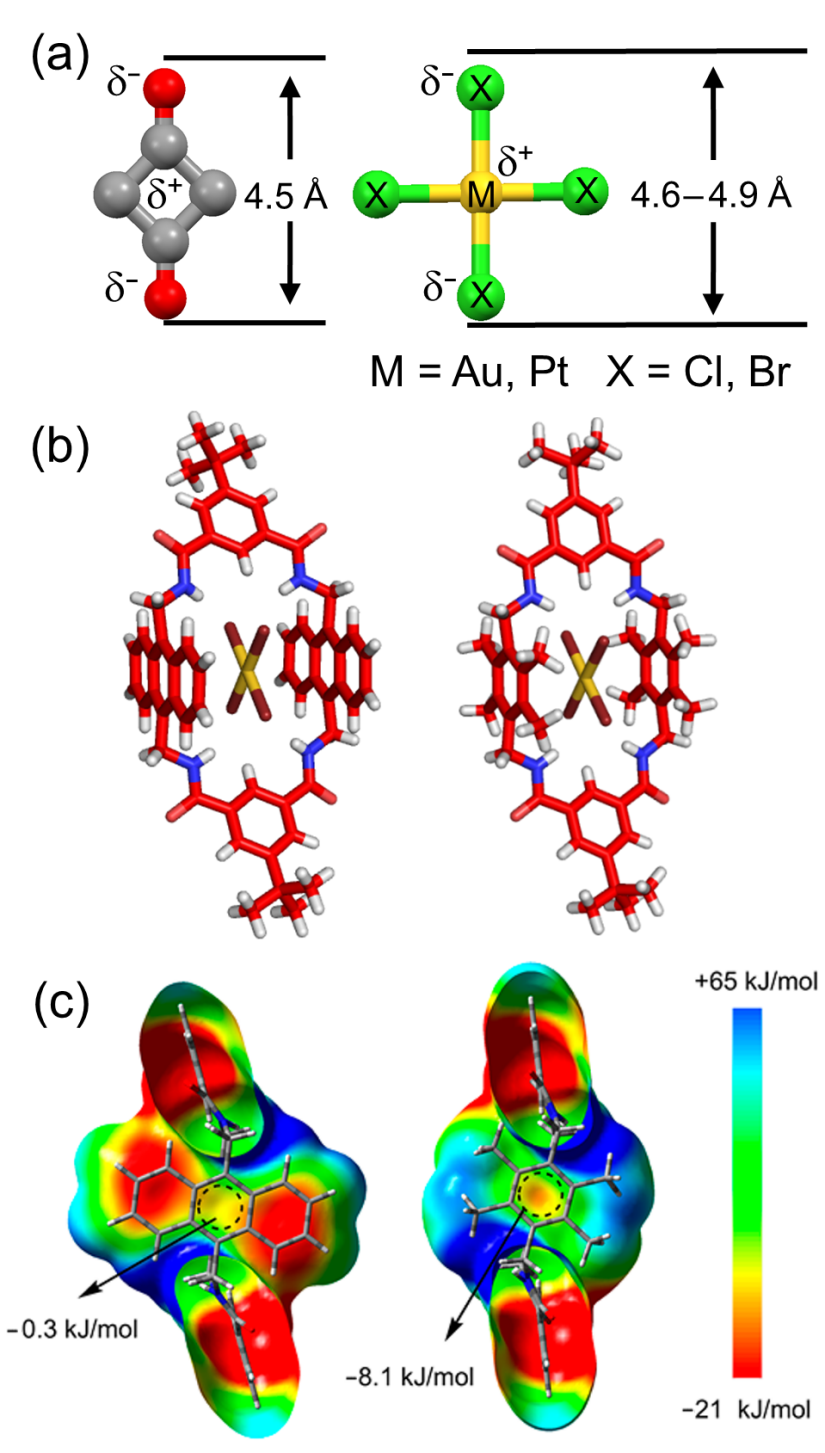
(a) Shape and electrostatic comparison of squaraine C_4_O_2_ core (left) with anionic square planar metal halide complexes (right). (b) Solid-state structures of **B**·AuBr_4_^−^ (X = CH, Z = *t*-Bu, left) and **C**·AuBr_4_^−^ (X = CH, Z = *t*-Bu, right). (c) Electrostatic potential maps of the interior surfaces of (left) **B** and (right) **C** obtained by DFT calculations at the B3LYP/631G* level. Reprinted with permission from [24], copyright 2018, American Chemical Society.

The electrostatic interior surface of the amphiphilic tetralactam cavity is complementary to the electrostatic surface of the core of a squaraine dye which has a partial positive charge at the center and a partial negative charge on the peripheral oxygens. The Smith group searched for other molecules with electrostatic shapes that are complementary to the tetralactam cavity (Figure 5a). They discovered that square planar precious metal halogen complexes such as AuCl_4_^−^, AuBr_4_^−^ and PtCl_4_^−^, are excellent guests [24]. Shown in Figure 5b are X-ray structures of AuBr_4_^−^ inside organic-soluble versions of tetralactam **B** and **C** with 9,10-anthrylene or 2,3,5,6-tetramethyl-1,4*-*phenylene sidewalls, respectively. Solution-state binding studies in organic solvents showed that tetralactam **C** exhibited higher affinity for the MX_4_^−^ complexes. The main reason for this difference is revealed by comparing the electrostatic surface maps of each cavity interior. The center of sidewalls in **C** is calculated to be 7.8 kJ mol^−1^ more negative than **B** and thus expected to interact more strongly with the metal center (Figure 5c). In addition, the peripheral methyl groups on each sidewall in **C** provide stabilizing CH···X interactions.

#### 3.4. Ammonium chloride ion pairs

**Figure 6:**
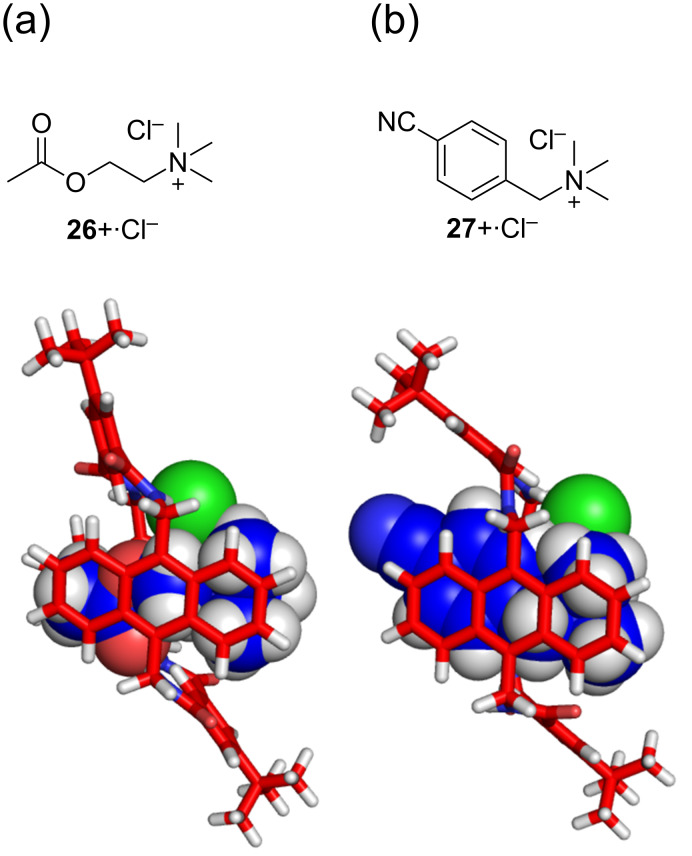
Chemical structures of a) acetylcholine chloride, **26**^+^·Cl^−^, (b) trimethyl-*p*-cyanobenzylammonium chloride, **27**^+^·Cl^−^, and calculated structures (semiempirical, PM7) of their complexes inside tetralactam **B** (X = CH, Z = *t*-Bu).

Another advantage of the amphiphilic macrocyclic cavity was recently uncovered by studies that showed simultaneous binding of tetralkylammonium chloride ion pairs, such as acetylcholine chloride, **26**^+^·Cl^−^, by tetralactam **B** [64]. As shown in Figure 6, the cavity can nicely accommodate ion pairs that can simultaneously contact the NH residues and the interior aromatic surfaces. The cavity was an especially good fit for trimethylbenzylammonium chloride salts where the guest benzyl group engages in aromatic stacking with the host anthracene sidewalls. Furthermore, the affinities followed a rough linear free energy relationship with electron density on the benzyl group, with highest affinity achieved when the benzylammonium contained a withdrawing *p*-CN group (i.e., **27**^+^·Cl^−^). Not only was **27**^+^·Cl^−^ a high affinity guest for tetralactam **B**, it was also an effective template for the macrocyclization reaction that produced tetralactam **B**.

#### 3.5. Oligosaccharides

**Figure 7:**
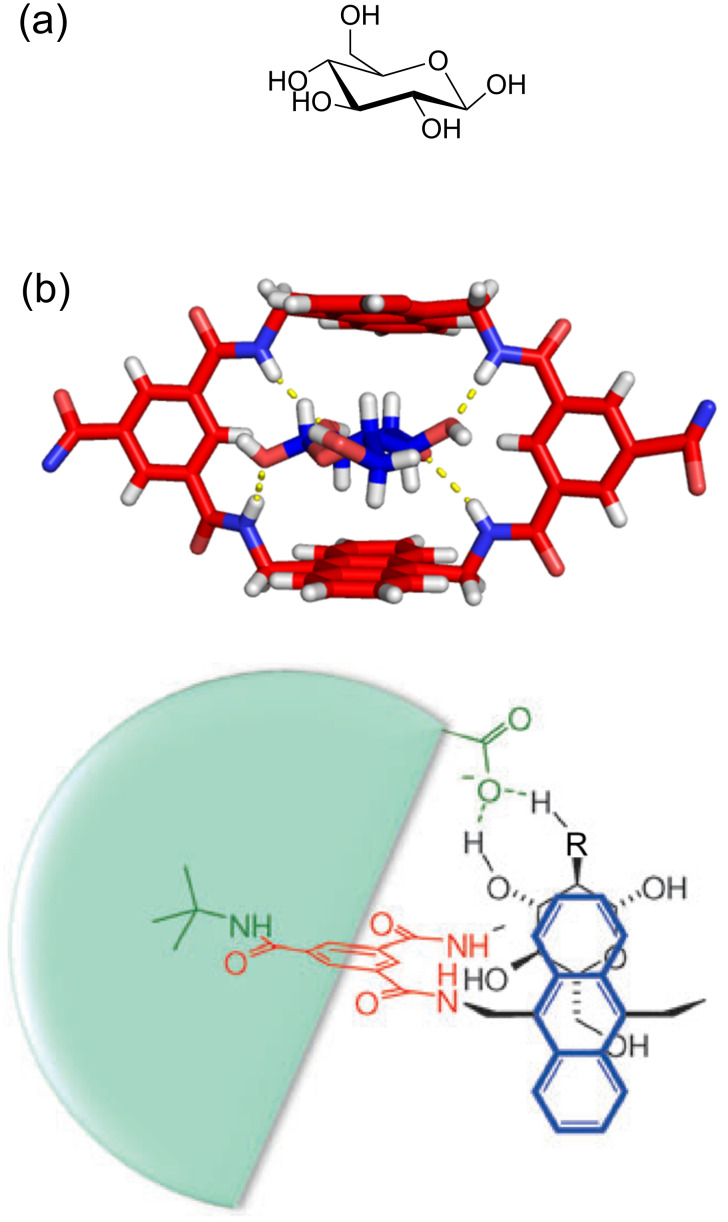
a) Chemical structure of β-D-glucopyranose and the solid-state structure of its complexes with macrocycle **B** (X = CH). b) Schematic view of tetralactam **B** encapsulating glucose (R = O) or glucosammonium (R = NH_2_^+^) with stabilizing secondary interactions provided by back-folding of a peripherally appended, anionic Z group. Adapted with permission from [71], copyright 2015, John Wiley and Sons.

The advantages of the amphiphilic cavity are also highlighted by the work of the Davis group who have shown that members of this tetralactam family are effective receptors for saccharides in water [65]. As shown by the X-ray crystal structure in Figure 7a, water-soluble versions of tetralactam **B** can nicely accommodate β-glucopyranose within the cavity [66]. There is a combination of hydrogen bonding between the equatorial sugar hydroxy groups and the receptor NH residues and also in the CH···π interactions with the hydrophobic anthrylene sidewalls. This combination of noncovalent interactions closely mimics the sugar-binding behavior exhibited by lectin proteins. The Davis group has explored more elaborate lactam-based receptors with biphenyl and pyrenyl sidewalls [67–69], and also the tetralactam version **F** which has eight extra methoxy groups attached to the anthrylene sidewalls [70]. The methoxy groups provide the sidewalls with increased electron density, which leads to tighter CH···π interactions and higher monosaccharide affinities. In addition, there was a remarkable improvement in affinity for larger oligosaccharides, presumably due to a stronger hydrophobic effect elicited by the extended hydrophobic surface of the tetralactam sidewalls. The Davis work has also uncovered a binding-enhancement effect that is analogous to the squaraine guest back-folding described above. In this present case, the anionic Z groups that are peripherally appended to a tetralactam host are long enough to fold back and provide stabilizing secondary interactions with a cationic glucosammonium guest inside the cavity [71].

## Conclusion

The binding pockets within enzymes, lectins, and related protein receptors are amphiphilic; that is, they contain a mixture of polar and non-polar functional groups. The macrocyclic tetralactams highlighted in this review are biomimetic in that they are synthetic host molecules with amphiphilic cavities. The non-polar aromatic surfaces in the host cavity drive high affinity in water due to the hydrophobic effect with the polar NH groups providing secondary interactions that induce binding selectivity. A practical advantage with these synthetic hosts is that they can be easily modified to be soluble in organic solvents, where the amphiphilic nature of the host cavity is again revealed, but for the reverse supramolecular reasons. The polar NH groups drive high affinity in organic solvents and the aromatic surfaces provide the secondary interactions. The moderate conformational flexibility of the macrocyclic tetralactams enables them to be effective hosts for a wide range of guest molecules including organic biscarbonyl derivatives, near-infrared dyes, acenes, precious metal halide complexes, trimethylammonium ion pairs, and saccharides. Because of this versatility, macrocyclic tetralactams with parallel aromatic sidewalls are well-suited for various practical supramolecular applications such as molecular machines [72], optical imaging [73], organocatalysis [55], detection [74], and separations [24].
